# Energy and Micronutrient Intakes Before and After Bariatric Surgery: Nutritional Challenges During the First Postoperative Year

**DOI:** 10.3390/nu18213549

**Published:** 2026-10-28

**Authors:** Karolina Brzostek, Jakub Krawczyk, Anna Jeznach-Steinhagen, Dorota Szostak-Węgierek, Rafał Marszałek, Iwona Boniecka

**Affiliations:** 1 Department of Clinical Dietetics, Faculty of Health Sciences, Medical University of Warsaw, 01-445 Warsaw, Poland; karolina.brzostek@wum.edu.pl (K.B.); jakub.krawczyk@wum.edu.pl (J.K.); anna.jeznach-steinhagen@wum.edu.pl (A.J.-S.); dorota.szostak-wegierek@wum.edu.pl (D.S.-W.); 2 Department of General Surgery, Military Institute of Medicine-National Research Institute, Zegrzyńska 8, 05-119 Legionowo, Poland; rmarszalek@wim.mil.pl

**Keywords:** bariatric surgery, sleeve gastrectomy, Roux-en-Y gastric bypass, dietary intake, weight loss, micronutrient intake

## Abstract

Background/Objectives: Metabolic/bariatric surgery (MBS) markedly reduces food intake, but longitudinal data on food-derived micronutrient intake during the first postoperative year are limited. This study aimed to characterize changes in dietary energy and micronutrient intake before and after sleeve gastrectomy (SG) and Roux-en-Y gastric bypass (RYGB). Methods: Seventy-three patients undergoing MBS (47 SG, 26 RYGB) were included. Dietary intake was assessed using a 24-h dietary recall before surgery and at 1, 6, and 12 months postoperatively. Energy and 12 micronutrients were analyzed using generalized estimating equations adjusted for sex, age, and baseline BMI, with Benjamini–Hochberg correction for multiple testing. Results: Intakes of energy and all micronutrients changed significantly over time (all adjusted *p* < 0.001) and remained below preoperative values throughout follow-up. For most nutrients, intake was lowest at 1 month and increased only partially by 12 months. No significant main effects of surgical procedure were observed, whereas measurement-by-procedure interactions were significant for energy and zinc. At 12 months, food-derived intake reached only 41–64% of the reference values for calcium, magnesium, iron, and folate. Conclusions: Food-derived energy and micronutrient intakes remained substantially reduced throughout the first postoperative year, supporting continued dietary monitoring alongside guideline-based supplementation and biochemical surveillance after bariatric surgery.

## 1. Introduction

Obesity is a chronic, progressive disease associated with type 2 diabetes, cardiovascular disease, impaired quality of life, and premature mortality. Its prevalence has risen markedly worldwide [1]. Metabolic/bariatric surgery (MBS) reduces food intake and improves obesity-related metabolic complications, but may also increase the risk of inadequate vitamin and mineral intake [2]. Nutritional assessment and follow-up are therefore essential components of postoperative care.

Bariatric procedures can compromise nutrient intake and micronutrient status by reducing food intake, decreasing gastric acid secretion, and altering food tolerance and eating behavior. After RYGB, bypass of the duodenum and proximal jejunum also affects absorption [3,4]. Postoperative nausea and vomiting, changes in appetite and taste, and poor adherence to lifelong supplementation may further increase nutritional risk [4]. Micronutrients of particular concern include iron, vitamin B12, folate, calcium, vitamin D, thiamine, zinc, copper, and fat-soluble vitamins [4,5].

During the first postoperative year, rapid weight loss coincides with changes in gastrointestinal anatomy, food tolerance, and eating behavior. Energy and nutrient intake usually fall sharply in the early postoperative period and recover only partially as the diet progresses [6]. The consequences differ between nutrients. Thiamine depletion can develop rapidly during persistent vomiting or very low intake, whereas deficiencies of iron, vitamin B12, calcium, and fat-soluble vitamins tend to develop or worsen more gradually [3,4,5]. These deficiencies may contribute to anemia, secondary hyperparathyroidism, bone loss, and neurological complications. They may persist or accumulate over years despite routine supplementation and follow-up [7,8,9,10].

Most studies of nutritional complications after bariatric surgery have assessed biochemical micronutrient status. Fewer have followed dietary vitamin and mineral intake over time or directly compared trajectories after SG and RYGB [6,11]. Dietary assessment can identify potentially modifiable gaps in food-derived intake before biochemical abnormalities become apparent. Repeated assessments are needed to capture the transition from early dietary restriction to greater food volume and variety during recovery.

The primary aim of this study was to characterize changes in food-derived energy and selected micronutrient intake before surgery and at 1, 6, and 12 months after SG and RYGB. Secondary aims were to compare intake trajectories between procedures and examine associations with postoperative weight-loss outcomes. Analyses addressing these secondary aims were considered exploratory.

## 2. Materials and Methods

### 2.1. Study Design and Participants

This longitudinal observational study included 73 adults who underwent MBS at the Military Institute of Medicine in Warsaw, Poland: 47 underwent SG and 26 underwent RYGB. Recruitment took place between August 2007 and December 2008, and the last 12-month follow-up visit occurred in 2009. Data were collected prospectively according to a standardized protocol within a project funded by the Ministry of Science and Higher Education (grant no. N N403 0336 33). The current investigation is a secondary analysis of these prospectively collected data, examining dietary intake trajectories and differences between procedures.

Participants were eligible if they had a BMI of 35–40 kg/m^2^ and obesity-related comorbidities likely to benefit from weight reduction, or a BMI of ≥40 kg/m^2^ regardless of comorbidities. These criteria were consistent with the reimbursement criteria of the Polish National Health Fund (NHF).

Exclusion criteria included severe or incurable diseases associated with cachexia, acute life-threatening conditions, uncompensated endocrine disorders responsible for obesity, severe coagulation disorders, active alcohol or drug dependence, uncontrolled psychiatric disorders, severe cognitive impairment, inability to participate in long-term follow-up, pregnancy or lactation, planned pregnancy within 12 months, and lack of informed consent.

Dietary and anthropometric assessments were performed before surgery and during postoperative follow-up at 1, 6, and 12 months.

The initial study size reflected the number of patients who attended the clinic during the recruitment period and initially expressed willingness to participate. No a priori sample-size calculation was performed for the present secondary analysis.

**Figure A1:**
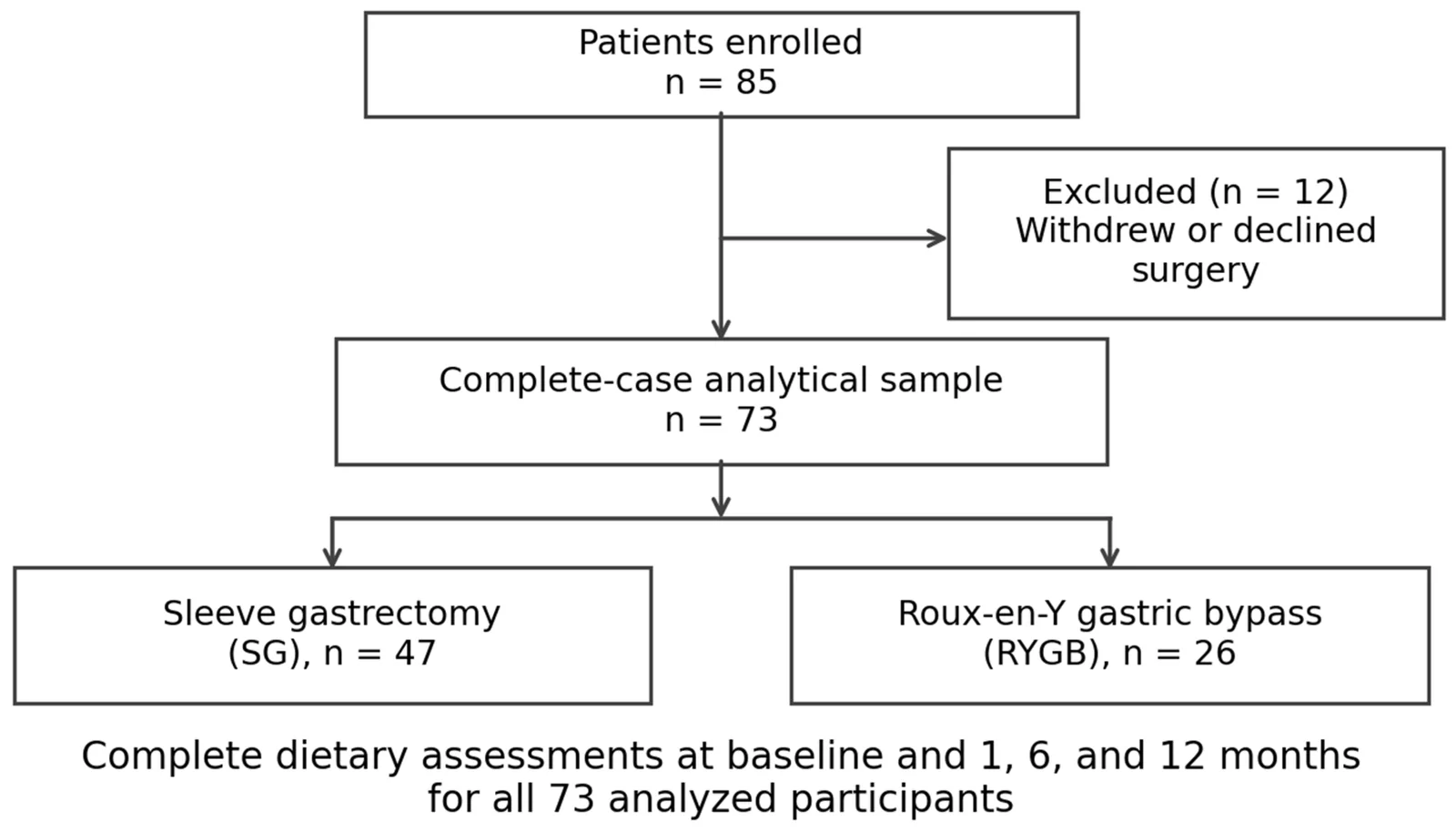
Participant flow and complete-case analytical sample. Of 85 enrolled patients, 12 withdrew or decided not to undergo surgery; 73 participants completed all four dietary assessments.

Only participants with complete dietary assessments at all four time points were included in the present analysis. Participants with incomplete dietary follow-up were excluded from the analytical sample. Of the 85 patients enrolled, 12 (14.1%) were not included in the analysis because they withdrew from the study or decided not to undergo surgery, leaving 73 participants (47 SG, 26 RYGB) with complete dietary data at all four assessments (Figure A1). Because only complete cases were analyzed, the GEE models contained no missing observations.

### 2.2. Bariatric Surgical Procedures

The surgical procedures included laparoscopic sleeve gastrectomy and laparoscopic Roux-en-Y gastric bypass. The type of surgical procedure was selected jointly by the surgeon and the patient following clinical assessment and dietetic consultation.

In SG, the lateral portion of the stomach was resected to create a narrow gastric tube along the lesser curvature, resulting in a substantial reduction in gastric volume [12]. In addition to its restrictive effect, SG is associated with changes in appetite regulation, including reduced ghrelin secretion.

RYGB involved the creation of a small gastric pouch connected to the small intestine, bypassing the proximal gastrointestinal tract [13,14]. In the surgical protocol, approximately 75–150 cm of proximal small intestine was bypassed, with an alimentary limb of approximately 100–150 cm. The procedure therefore combines restriction of food intake with altered nutrient absorption and gastrointestinal hormone secretion.

### 2.3. Dietary Intake Assessment

Dietary intake was assessed using a 24-h dietary recall. During dietary interviews, participants reported all foods and beverages consumed during the preceding 24 h, including the timing and estimated portion size of individual meals.

Portion sizes were estimated with the aid of the Album of Photographs of Food Products and Dishes [15]. A dietitian reviewed the recalls, and energy and nutrient intakes were calculated using Diet 5 software (National Food and Nutrition Institute, Warsaw, Poland, 2011).

Dietary assessments were conducted before surgery and at 1, 6, and 12 months postoperatively. The analysis included nutrients from foods and beverages only. Postoperative dietary management followed a staged protocol consistent with the nutritional principles of the AACE/TOS/ASMBS guidelines [16], with progression adapted to the procedure and individual tolerance. After both SG and RYGB, clear liquids were introduced during the first two postoperative days, followed by full liquids from approximately day 3. Puréed and soft foods were introduced around postoperative days 10–14, with further expansion during weeks 4 and 5 and progression to a balanced solid-food diet as tolerated. Patients were advised to eat small, frequent meals, chew thoroughly, maintain adequate hydration, separate food and fluid intake, and aim for 60–80 g of protein per day. Individual dietary counseling was provided at the 1-, 6-, and 12-month visits. Routine vitamin and mineral supplementation was recommended, but actual use, products, doses, and adherence were not recorded.

### 2.4. Anthropometric Measurements and Weight-Loss Outcomes

Body weight was measured using a Tanita TBF-300P scale (Tanita Corporation, Tokyo, Japan), and height was measured using a wall-mounted SECA 216 stadiometer (SECA, Hamburg, Germany). Body mass index (BMI) was calculated as body weight in kilograms divided by height in meters squared.

Postoperative weight-loss outcomes were evaluated using percentage excess weight loss (%EWL) and percentage total weight loss (%TWL).

Percentage excess weight loss was calculated according to the following formula [17]:

%EWL = [(preoperative weight − postoperative weight)/(preoperative weight − ideal weight)] × 100

where ideal body weight corresponded to a BMI of 25 kg/m^2^.

Percentage total weight loss was calculated as:

%TWL = [(preoperative weight − postoperative weight)/preoperative weight] × 100

Both %EWL and %TWL were analyzed as time-varying measures during postoperative follow-up.

### 2.5. Assessment of Energy and Micronutrient Intake

The analysis covered daily energy intake and selected vitamins and minerals relevant to nutritional care after MBS.

The following variables were evaluated: energy (kcal/day), calcium (mg/day), magnesium (mg/day), iron (mg/day), zinc (mg/day), vitamin A (µg/day), vitamin E (mg/day), thiamine (vitamin B1; mg/day), riboflavin (vitamin B2; mg/day), niacin (vitamin PP; mg/day), vitamin B6 (mg/day), folate (µg/day), and vitamin B12 (µg/day).

Nutrient intake was evaluated at four time points: before surgery and at 1, 6, and 12 months postoperatively.

Food-derived nutrient intakes were compared descriptively with the Nutrition Standards for the Polish Population (2020) [18]. Each participant was assigned age- and sex-specific recommended dietary allowance (RDA) values, or adequate intake (AI) values for vitamin E. For iron, women aged ≥51 years were assigned the RDA specified for that age group. Individual reference values were averaged within each surgical group. Mean intakes at each assessment were expressed as percentages of the corresponding group reference values. The proportion of participants whose intake on the day covered by the recall reached or exceeded their individual RDA or AI was also calculated.

### 2.6. Statistical Analysis

Changes in energy and micronutrient intake across the four assessments were analyzed using generalized estimating equations (GEE) with an autoregressive working correlation structure, a gamma distribution, and a log link. Robust sandwich standard errors were used for inference. The correlation structure was selected to allow stronger correlations between successive assessments than between assessments further apart. The working correlation matrix was updated at each iteration with maximum number of iterations equal to 100. The iterations allowed for adjusting the preliminary correlation matrix and were necessary, because the consecutive measurements were not equally spaced in time. Separate models were fitted for each of the 13 outcomes: energy, calcium, magnesium, iron, zinc, vitamin A, vitamin E, thiamine, riboflavin, niacin, vitamin B6, folate, and vitamin B12. Each model included measurement time (Meas.), surgical procedure (Group; SG vs. RYGB), and their interaction (Meas. × Group), with adjustment for sex, age, and baseline BMI. Estimated marginal means and 95% confidence intervals were reported for each procedure and assessment. Model effects were evaluated using Wald chi-square statistics, degrees of freedom, and *p*-values. For significant interaction effects, Cohen’s d was calculated as the difference between estimated marginal means at consecutive assessments divided by the pooled raw standard deviation and not on the standard deviation of paired differences.

To account for multiple comparisons, *p*-values were adjusted using the Benjamini–Hochberg procedure. The correction was applied separately across the 13 nutrients outcomes for each model effect, i.e., time, procedure, and the interaction between the two.

%EWL and %TWL were examined separately as time-varying covariates in additional GEE models, controlling for measurement time and surgical procedure. Regression coefficients (B), Wald chi-square statistics, degrees of freedom, and *p*-values were reported. Analyses were performed using IBM SPSS Statistics version 32.0.

### 2.7. Ethical Considerations

All participants received information regarding the study procedures and provided written informed consent prior to participation. The study was conducted in accordance with the Declaration of Helsinki and approved by the Bioethics Committee of the Medical University of Warsaw (approval nos. KB/126/2006 and KB/66/A/2007).

## 3. Results

### 3.1. Characteristics of the Study Population

The analytical sample comprised 73 participants: 47 in the SG group and 26 in the RYGB group. The SG group included 29 women and 18 men, aged 23–65 years (mean 44.21 ± 12.30 years). The RYGB group included 22 women and 4 men, aged 22–53 years (mean 35.42 ± 9.21 years). Overall mean age was 41.08 ± 12.00 years. Baseline characteristics are summarized in Table 1.

In the SG group, BMI ranged from 39.51 to 66.29 kg/m^2^, with a mean of 49.73 ± 7.16 kg/m^2^. In the RYGB group, BMI ranged from 35.25 to 51.20 kg/m^2^, with a mean of 42.40 ± 4.81 kg/m^2^.

All 73 participants completed all four dietary assessments (Figure A1). Sex distribution differed between groups (Pearson’s chi-square test, χ^2^(1) = 4.17, *p* = 0.041). Independent-samples *t*-tests also showed differences in age (*t*(71) = 3.18, *p* = 0.002) and baseline BMI (*t*(71) = 4.66, *p* < 0.001). Sex, age, and baseline BMI were included as covariates in subsequent analyses.

### 3.2. Changes in Energy and Micronutrient Intake Across Measurements

Statistically significant effects of measurement were observed for all 13 analyzed variables (Benjamini–Hochberg-adjusted *p* < 0.001). Energy and nutrient intakes were lower after surgery.

For most nutrients, mean intake was lowest at 1 month and increased by 12 months but remained well below preoperative values (Table A1).

The estimated marginal means and 95% confidence intervals for all outcomes at each assessment are presented in Table A1 in Appendix A.

### 3.3. Effects of Surgical Procedure and Measurement-by-Procedure Interactions

After adjustment for multiple testing, no statistically significant main effects of surgical procedure were observed.

Significant measurement-by-procedure interactions were observed for energy and zinc intake (Benjamini–Hochberg-adjusted *p* = 0.013 for each outcome).

**Figure 1:**
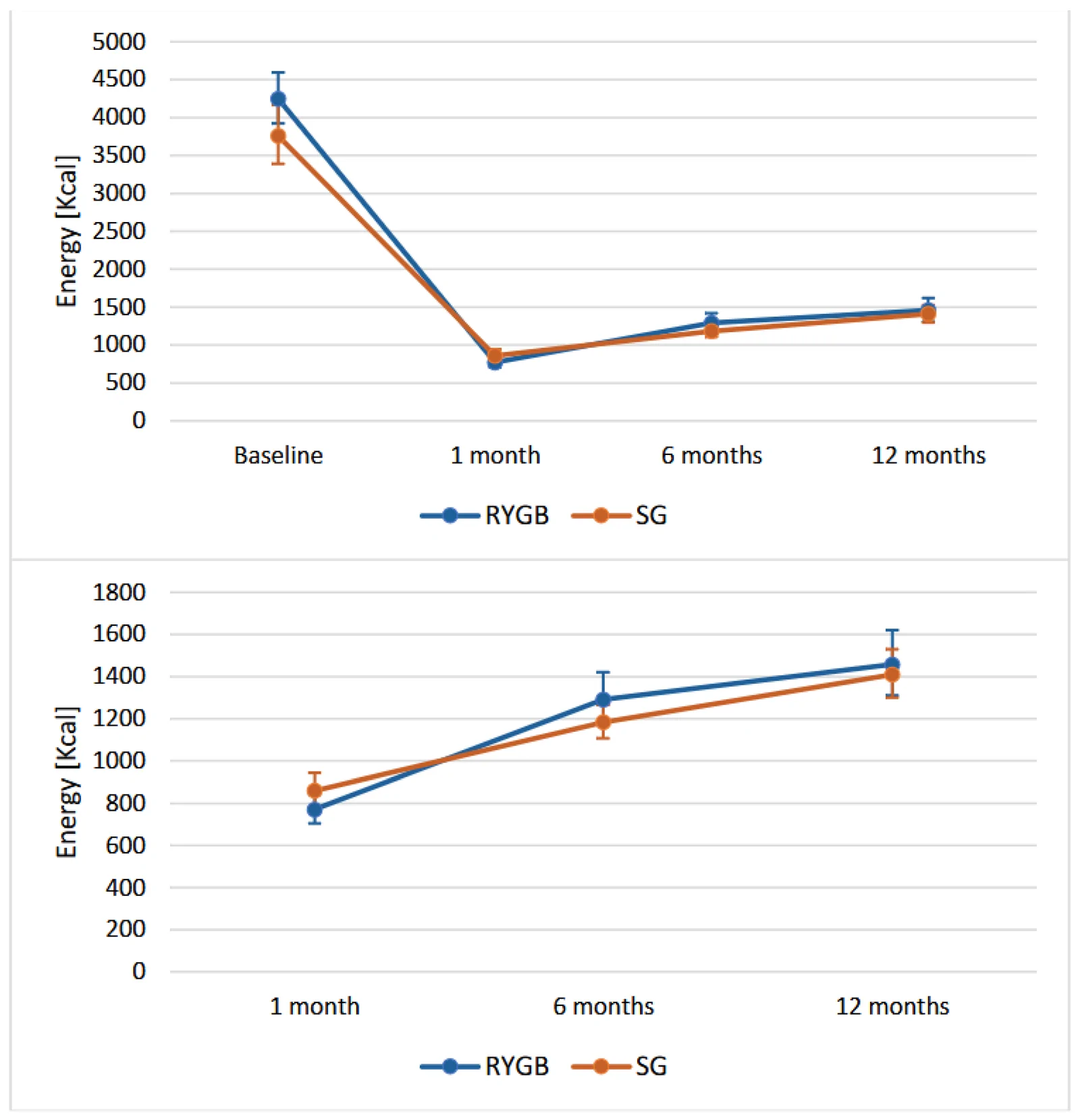
Food-derived energy intake by surgical procedure across the four assessments. The second panel shows postoperative assessments only. Points represent estimated marginal means and error bars indicate 95% confidence intervals.

**Figure A2:**
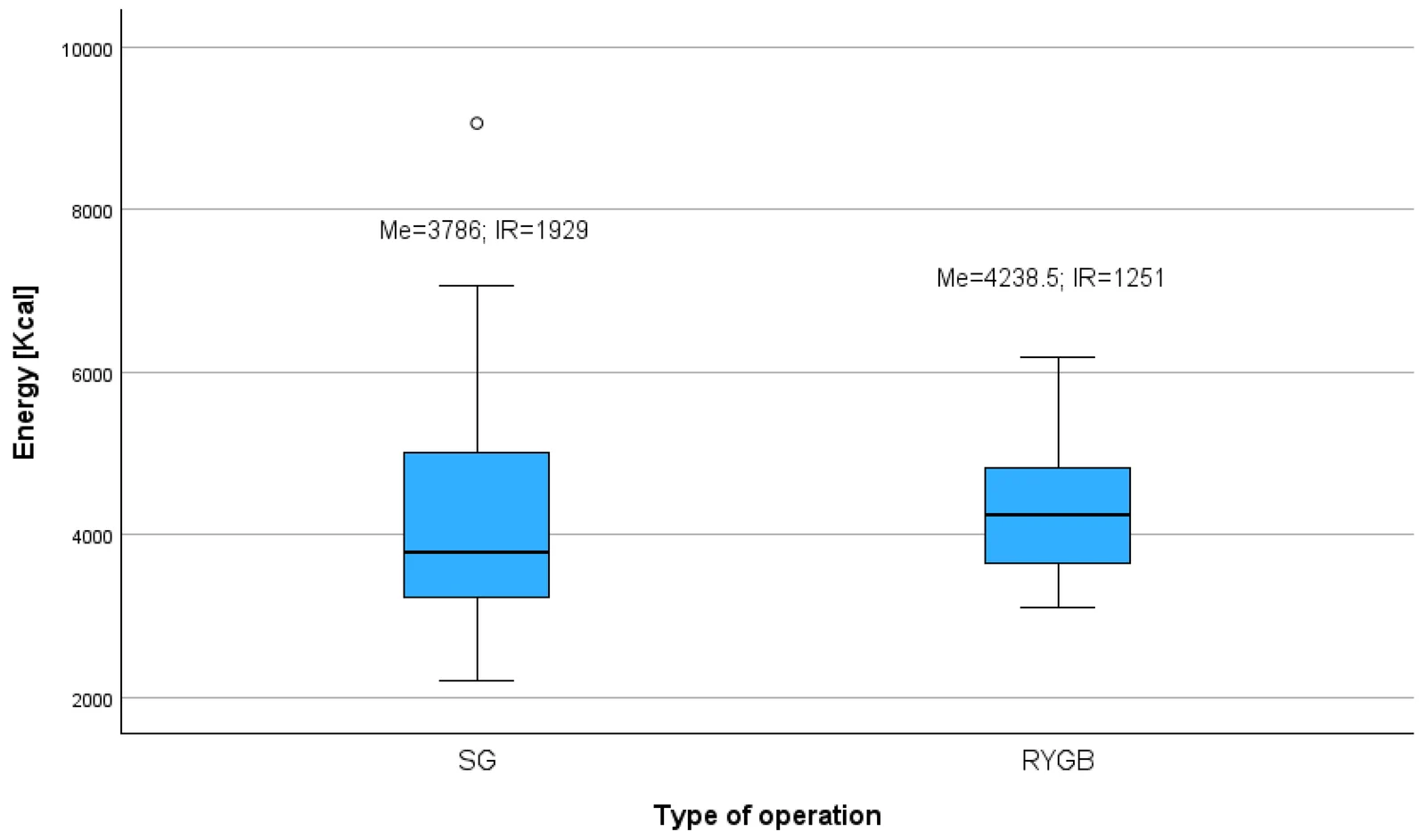
Distribution of preoperative food-derived energy intake by surgical procedure. SG, sleeve gastrectomy; RYGB, Roux-en-Y gastric bypass; Me, median; IR, interquartile range.

For energy intake, the standardized reduction from baseline to 1 month was d = 1.75 in the RYGB group and d = 1.51 in the SG group (Figure 1). The distribution of preoperative energy intake in both groups is shown in Figure A2 in Appendix A. One outlier in the SG group was detected. However, the interaction effect was also statistically significant after removing the observation from the sample, χ^2^(3) = 14.23, *p* = 0.003.

**Figure 2:**
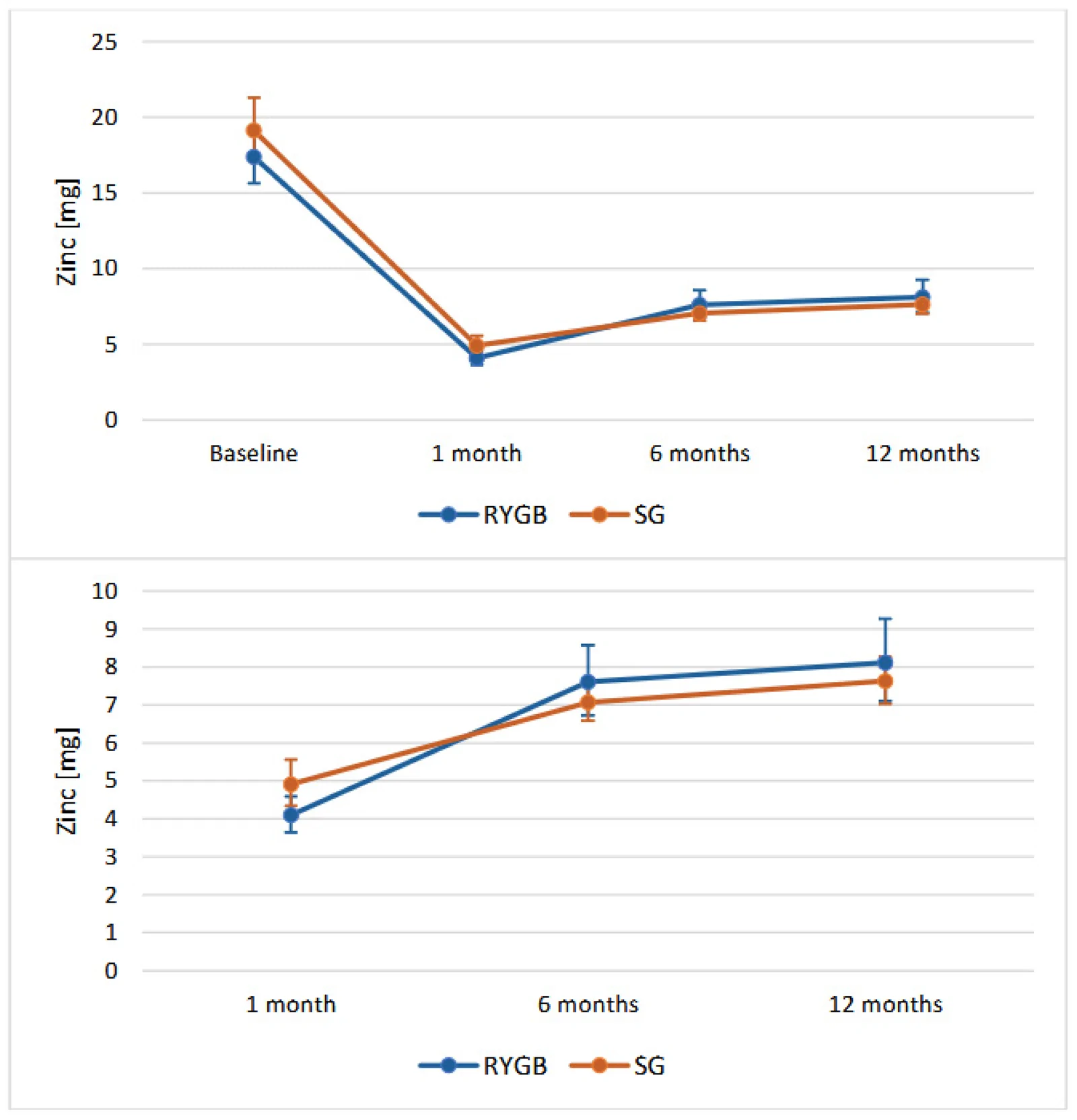
Food-derived zinc intake by surgical procedure across the four assessments. The second panel shows postoperative assessments only. Points represent estimated marginal means and error bars indicate 95% confidence intervals.

For zinc intake, the corresponding standardized reductions were d = 1.81 in the RYGB group and d = 1.46 in the SG group (Figure 2).

No statistically significant measurement-by-procedure interactions were observed for the remaining analyzed variables.

### 3.4. Associations with Weight-Loss Outcomes

Percentage excess weight loss and percentage total weight loss were examined separately as time-varying covariates in GEE models, with measurement time and surgical procedure retained in each model (Table 2). These analyses were considered exploratory.

In the additional exploratory models, the estimated coefficient for energy intake was negative for %EWL (B = −0.01).

These estimates describe associations within the specified models and do not establish a causal effect of dietary intake on weight loss.

### 3.5. Descriptive Comparison with Dietary Reference Values

At 12 months, mean food-derived intake expressed relative to the group reference value was 52–64% for calcium, 58–60% for magnesium, 48–63% for iron, 83–87% for zinc, 71–75% for thiamine, 82–84% for niacin, 109–112% for vitamin B6, 121–131% for vitamin B12, and 41–43% for folate across the two surgical groups (Table 3), whereas the corresponding values for vitamin A, vitamin E, and riboflavin were 97–104%, 85–110%, and 109–117%, respectively. On the recall day, no more than 15% of participants reached their individual reference value for calcium, 4% for magnesium, 19% for iron, and 2% for folate; fewer than half reached their reference value for zinc. These descriptive comparisons refer to the assessed day and do not estimate the prevalence of inadequate usual intake.

## 4. Discussion

Energy and micronutrient intakes decreased after SG and RYGB. For most nutrients, intake was lowest at 1 month and recovered only partially by 12 months. After adjustment for multiple testing, no main effects of procedure were statistically significant; time-by-procedure interactions were observed for energy and zinc. The time-by-procedure interaction for energy remained statistically significant after exclusion of the identified outlier in the SG group. The early decline is consistent with the small food volumes consumed during progression from liquids to solid foods, alongside changes in appetite, satiation, food preferences, and gastrointestinal tolerance [3,4,5,6].

Similar patterns have been reported in longitudinal studies. Gesquiere et al. found that dietary iron, vitamin B12, vitamin C, copper, and zinc intake decreased at 1 month after RYGB and subsequently increased, but remained below baseline at 12 months. When supplements were included, total intake exceeded baseline for most nutrients except zinc [19]. Heusschen et al. reported lower energy and micronutrient intake and changes in diet quality six months after surgery [20]. A systematic review found that postoperative diets were often unbalanced, with inadequate micronutrient and protein intake and relatively high fat intake [21]. Ziadlou et al. reported dietary inadequacy at 6 and 12 months, with no participant achieving adequate dietary zinc intake [22]. A meta-analysis of 42 studies by Qanaq et al. found reduced postoperative energy intake, no consistent overall difference between SG and RYGB, and frequently inadequate iron, calcium, and zinc intake [23]. Our study extends these observations by following a broad panel of food-derived micronutrients at four assessments before and after both procedures.

In the randomized Bypass Equipoise Sleeve Trial (BEST) substudy, 285 participants were assessed one year after SG or RYGB. Energy and micronutrient intake did not differ significantly between procedures, although several micronutrients remained below recommended levels. Total intake from food and supplements differed substantially from dietary intake alone [24]. Energy intake at 12 months in our cohort was lower than the approximately 1650 to 1670 kcal/day reported in BEST. This comparison is limited by differences in assessment methods: BEST used a four-day food record with Goldberg cut-offs, whereas we used a single 24-h recall. Both methods rely on self-report and are susceptible to measurement error and misreporting [24,25,26].

Calcium intake remained below baseline at every postoperative assessment in both groups. Bariatric surgery can also affect bone health through changes in vitamin D metabolism, calcium absorption, hormonal regulation, and the skeletal effects of rapid weight loss [4,5,9,10]. A meta-analysis reported greater reductions in bone mineral density after RYGB than after SG, together with differences in vitamin D and parathyroid hormone concentrations [27]. Dietary calcium assessment should therefore be considered alongside biochemical monitoring [4,28]. The low food-derived intake reported here does not establish calcium deficiency.

Iron intake was lowest at 1 month and increased thereafter, but remained below baseline. Reduced consumption of iron-rich foods, lower gastric acidity, and bypass of the proximal small intestine after RYGB may compromise iron status [3,4,5]. SG also carries a risk of iron deficiency, and biochemical assessment is needed after both procedures [4,28]. A meta-analysis of randomized trials found no significant difference between SG and RYGB in iron or folate deficiency risk, although vitamin B12 deficiency was more frequent after RYGB [29]. Anemia and iron-related abnormalities may persist for years after surgery [7,8]. Our findings support attention to dietary iron sources alongside laboratory monitoring.

Zinc and energy were the only outcomes with significant time-by-procedure interactions. The reduction in zinc intake from baseline to 1 month was larger after RYGB when expressed as Cohen’s d (1.81 vs. 1.46 after SG). The small number of patients, particularly in the RYGB group, limits the conclusions about differences between procedures. A 2026 meta-analysis of 12 studies involving 2529 participants reported reductions in serum zinc at 6 and 12 months, with significant reductions after RYGB but not SG [30]. Serum zinc and dietary zinc intake measure different aspects of nutritional status, so those findings cannot directly explain our results. Smaller portions of zinc-rich foods, including meat, seafood, dairy products, nuts, and seeds, and changes in food tolerance may contribute to lower dietary intake.

Thiamine intake decreased after surgery and remained below the descriptive reference value at 12 months. Limited body stores make thiamine deficiency a concern during very low intake or recurrent vomiting [3,4,5]. A meta-analysis estimated deficiency prevalence at approximately 19%, 9%, and 6% at 3, 6, and 12 months, respectively, with substantial heterogeneity [31]. Vitamin B12 intake also decreased, but mean food-derived intake exceeded the reference value at 12 months. This does not establish adequate vitamin B12 status, because postoperative changes can impair absorption independently of dietary supply [4,28]. The higher biochemical deficiency risk after RYGB reported in randomized studies [29] cannot be inferred from these dietary data. Folate showed the largest shortfall relative to the reference value among the vitamins examined at 12 months, supporting attention to food sources and routine monitoring [4,28].

For thiamine, niacin, vitamin B6, and vitamin B12, neither procedure effects nor time-by-procedure interactions were significant after adjustment for multiple testing. In the randomized Oseberg trial, food-derived vitamin and mineral intake decreased after both procedures, with greater reductions in some nutrients, including magnesium, after SG. Total intakes of several micronutrients increased when supplements were included [6]. BEST also reported broadly similar dietary intakes after SG and RYGB at one year [24]. In our descriptive comparisons, mean magnesium and niacin intake remained below their reference values at 12 months, whereas vitamin B6 intake was above its reference value.

The additional models examined %EWL and %TWL separately while controlling for measurement time and surgical procedure. Both measures are closely related, and both weight loss and dietary intake change during postoperative recovery. The observed associations remain exploratory: adjustment for time does not establish causality or eliminate residual confounding. Qanaq et al. likewise found no consistent association between postoperative energy intake and weight-loss outcomes [23].

Dietary counseling can help patients improve nutrient intake within the limits of postoperative food tolerance. However, diet alone is often insufficient to meet nutritional requirements, and care also requires guideline-based supplementation, laboratory monitoring, and long-term follow-up [4,5,32]. Côté et al. reported similar rates of several micronutrient deficiencies after SG and RYGB when supplementation was guided by procedure and laboratory results [33]. Food-derived intake is therefore one component of nutritional assessment and should be interpreted alongside supplement use and biochemical status.

The study followed food-derived intake at four time points during the first postoperative year, included both SG and RYGB, and assessed a broad range of micronutrients. GEE models accounted for repeated measurements, while additional analyses examined associations with weight-loss outcomes.

The principal limitation is the use of a single 24-h recall at each assessment. Recall bias, day-to-day variation, and dietary misreporting limit its ability to characterize individual usual intake. The proportions reaching RDA or AI values describe the recall day only and do not estimate the prevalence of inadequate usual intake. Studies using dietary records and objective methods such as doubly labeled water have documented limitations of self-reported energy intake, although recalls can provide useful information on micronutrient intake [24,25,26]. The high preoperative energy values should therefore be interpreted cautiously.

Supplement use was not recorded, and biochemical micronutrient measurements were unavailable. Supplements can contribute substantially to total postoperative intake [19,24], but we could neither estimate total intake nor account for differences in supplementation between procedures. Low food-derived intake cannot be interpreted as evidence of biochemical deficiency.

The groups were small and unequal in size (47 SG and 26 RYGB), and the RYGB group included only four men. Procedure selection was a joint decision by the surgeon and patient rather than randomized. Adjustment for sex, age, and baseline BMI does not exclude residual confounding. Precision was limited, particularly for interaction effects, and non-significant findings do not establish equivalence between procedures. Complete-case selection may also introduce bias if excluded participants differed from those analyzed. Of 85 enrolled patients, 12 (14.1%) withdrew or decided not to undergo surgery. The secondary analysis included all eligible complete cases, without an a priori sample-size calculation, and may have been underpowered to detect modest time-by-procedure interactions.

The single-center setting limits generalizability, and the one-year follow-up cannot show whether intake subsequently increases, stabilizes, or declines. Nutritional abnormalities may remain clinically relevant for many years after bariatric surgery [7,8,34].

The historical period of data collection (2007–2009) also limits comparison with current practice. Bariatric care has since changed through refinements in surgical technique, broader use of preoperative low-calorie diets, enhanced recovery protocols, updated supplementation guidelines [4,5,28], and perioperative incretin-based pharmacotherapy. The baseline recall describes one preoperative day and cannot establish usual intake or whether patients were following a low-calorie or liver-reduction diet. SG and RYGB remain widely performed procedures [35], and both continue to restrict food volume and alter food tolerance. The early decline and partial recovery observed here are also reported in more recent studies [19,23,24]. These historical data can inform understanding of intake trajectories, but should not be taken as estimates of absolute intake under current care protocols.

Future studies should combine repeated dietary assessments with supplement-use data and biochemical measurements. Multiple recalls or multi-day records would improve estimation of usual intake. Larger, more balanced surgical groups would support more precise procedure comparisons, while analysis of food sources could help guide postoperative counseling.

## 5. Conclusions

Food-derived energy and micronutrient intakes were lower after surgery than before surgery throughout the first postoperative year. For most nutrients, intake was lowest at 1 month and recovered only partially by 12 months.

After adjustment for multiple testing, no main effects of surgical procedure were statistically significant. Time-by-procedure interactions were observed for energy and zinc, indicating different intake trajectories across the four assessments.

Associations with weight-loss outcomes were exploratory and do not establish causality. Comparisons with dietary reference values describe intake on the recall day and do not determine usual-intake adequacy or diagnose micronutrient deficiency.

Postoperative nutritional care should combine dietary assessment and individualized counseling with appropriate supplementation and biochemical monitoring.

## Figures and Tables

**Table 1:** Baseline characteristics of the study participants by surgical procedure.

Characteristic	Total (n = 73)	SG (n = 47)	RYGB (n = 26)	*p*
Women, n (%)	51 (69.9)	29 (61.7)	22 (84.6)	0.041 ^a^
Men, n (%)	22 (30.1)	18 (38.3)	4 (15.4)	
Age, years, mean ± SD	41.08 ± 12.00	44.21 ± 12.30	35.42 ± 9.21	0.002 ^b^
Age, years, range	22–65	23–65	22–53	
BMI, kg/m^2^, mean ± SD	47.12 ± 7.30	49.73 ± 7.16	42.40 ± 4.81	<0.001 ^b^
BMI, kg/m^2^, range	35.25–66.29	39.51–66.29	35.25–51.20	

^a^ Pearson’s chi-square test, χ^2^(1) = 4.17; ^b^ independent-samples *t*-test, t(71) = 3.18 for age and t(71) = 4.66 for BMI. SG, sleeve gastrectomy; RYGB, Roux-en-Y gastric bypass; BMI, body mass index; SD, standard deviation.

**Table 2:** Percentage excess weight loss and percentage total weight loss as time-varying covariates in generalized estimating equation models.

Outcome	%EWL				%TWL			
	B	χ^2^	*df*	*p*	B	χ^2^	*df*	*p*
Energy	−0.01	11.81	1	0.013	−0.01	7.17	1	0.078
Calcium	−0.01	0.40	1	0.711	−0.01	0.09	1	0.905
Magnesium	−0.01	2.30	1	0.419	−0.01	1.94	1	0.529
Iron	−0.01	3.96	1	0.204	−0.01	2.39	1	0.529
Zinc	−0.01	5.29	1	0.137	−0.01	6.28	1	0.078
Vitamin A	0.01	0.02	1	0.891	−0.01	0.03	1	0.927
Vitamin E	−0.01	0.64	1	0.711	−0.01	0.73	1	0.691
Vitamin B1	0.01	0.34	1	0.711	−0.01	0.30	1	0.759
Vitamin B2	−0.01	0.99	1	0.693	−0.01	0.88	1	0.691
Niacin	0.01	0.27	1	0.711	0.01	0.01	1	0.927
Vitamin B6	−0.01	0.15	1	0.755	−0.01	0.36	1	0.759
Folate	−0.01	1.08	1	0.693	−0.01	0.77	1	0.691
Vitamin B12	0.01	0.38	1	0.711	0.01	0.64	1	0.691

B, interaction effect estimate (gamma distribution, log link; models included measurement time, surgical procedure, and their interaction); χ^2^, Wald chi-square statistic; df, degrees of freedom; *p*, *p*-value adjusted with Benjamini–Hochberg procedure; %EWL, percentage excess weight loss; %TWL, percentage total weight loss.

**Table 3:** Food-derived micronutrient intake in relation to the Nutrition Standards for the Polish Population (2020) by surgical procedure: observed mean intake as a percentage of the group reference value and percentage of participants whose intake on the recall day reached their individual reference value.

Micronutrient [Unit]	Group	Reference (RDA/AI)	% of Reference Value			% of Participants Reaching Reference		
			1 mo	6 mo	12 mo	1 mo	6 mo	12 mo
Calcium [mg]	RYGB	1015	46	61	64	4	8	15
	SG	1047	38	51	52	2	4	4
Magnesium [mg]	RYGB	332	37	54	60	0	0	0
	SG	356	43	54	58	2	0	4
Iron [mg]	RYGB	16.2	25	42	48	0	0	0
	SG	13.1	41	58	63	2	11	19
Zinc [mg]	RYGB	8.5	46	83	87	0	23	35
	SG	9.1	56	78	83	4	13	23
Vitamin A [µg]	RYGB	731	97	115	97	27	38	35
	SG	777	95	121	104	32	49	38
Vitamin E [mg]	RYGB	8.3	59	96	85	15	38	31
	SG	8.8	59	91	110	11	34	49
Thiamine (B1) [mg]	RYGB	1.1	38	63	71	0	12	23
	SG	1.2	48	60	75	6	6	13
Riboflavin (B2) [mg]	RYGB	1.1	79	109	117	23	62	69
	SG	1.2	90	106	109	28	55	57
Niacin [mg]	RYGB	14.3	49	74	82	4	15	31
	SG	14.8	74	74	84	15	15	21
Vitamin B6 [mg]	RYGB	1.3	73	98	109	19	46	54
	SG	1.4	86	105	112	28	47	57
Folate [µg]	RYGB	400	26	35	41	0	0	0
	SG	400	27	41	43	0	2	2
Vitamin B12 [µg]	RYGB	2.4	95	121	121	31	62	65
	SG	2.4	125	138	131	40	60	62

Values are observed (unadjusted) mean intakes expressed as a percentage of the group reference value, calculated as the mean of the participants’ individual age- and sex-specific recommended dietary allowances (RDA), or adequate intake (AI) for vitamin E, from the Nutrition Standards for the Polish Population (2020) [18]; for iron, women aged ≥51 years were assigned the RDA for that age group. Percentages of participants refer to food-derived intake on the single recall day and do not estimate the prevalence of inadequate usual intake. RDA, recommended dietary allowance; AI, adequate intake; SG, sleeve gastrectomy; RYGB, Roux-en-Y gastric bypass; mo, months.

## Data Availability

The data presented in this study are available upon request from the corresponding author. The data are not publicly available as they are still being used for analysis and manuscript writing.

## Abbreviations

MBS, metabolic/bariatric surgery; BMI, body mass index; %EWL, percentage excess weight loss; %TWL, percentage total weight loss; GEE, generalized estimating equations; RYGB, Roux-en-Y gastric bypass; SG, sleeve gastrectomy.

## Appendix A

**Table A1:** Longitudinal changes in food-derived energy and micronutrient intake by surgical procedure in generalized estimating equation models. Values are estimated marginal means [95% confidence intervals].

		Estimated Marginal Means [95% CI]				Main Effects				Interaction Effects		
Variable	Procedure	Baseline	1 Month	6 Months	12 Months		χ^2^	*df*	*p*	χ^2^	*df*	*p*
Energy [kcal]	RYGB	4243.21 [3920.81;4592.11]	770.16 [704.84;841.54]	1291.10 [1173.65;1420.31]	1457.10 [1310.56;1620.03]	Meas.	1323.73	3	<0.001	Meas. × Group		
	SG	3756.35 [3388.20;4164.51]	859.12 [782.04;943.79]	1183.07 [1106.69;1264.72]	1409.47 [1299.21;1529.08]	Group	0.53	1	0.864	14.71	3	0.013
Calcium [mg]	RYGB	1341.19 [1016.43;1769.71]	463.60 [373.86;574.87]	604.99 [446.63;819.49]	637.78 [472.87;860.21]	Meas.	76.85	3	<0.001	Meas. × Group		
	SG	1124.83 [846.97;1493.84]	402.72 [344.86;470.29]	529.88 [454.10;618.31]	536.42 [446.03;645.12]	Group	2.21	1	0.445	0.09	3	0.993
Magnesium [mg]	RYGB	475.63 [428.79;527.58]	126.83 [113.73;141.44]	190.33 [166.13;218.05]	212.35 [182.57;246.98]	Meas.	423.40	3	<0.001	Meas. × Group		
	SG	458.93 [400.72;525.59]	147.00 [126.04;171.44]	189.17 [173.78;205.91]	206.59 [184.92;230.80]	Group	0.09	1	0.877	3.16	3	0.532
Iron [mg]	RYGB	19.35 [17.03;21.99]	4.26 [3.85;4.71]	7.31 [6.28;8.50]	8.33 [6.85;10.12]	Meas.	558.87	3	<0.001	Meas. × Group		
	SG	19.19 [17.01;21.66]	5.17 [4.50;5.93]	7.44 [6.83;8.09]	8.25 [7.55;9.01]	Group	0.52	1	0.864	4.77	3	0.418
Zinc [mg]	RYGB	17.39 [15.64;19.34]	4.09 [3.64;4.59]	7.60 [6.73;8.58]	8.11 [7.10;9.27]	Meas.	714.67	3	<0.001	Meas. × Group		
	SG	19.13 [17.18;21.30]	4.91 [4.34;5.56]	7.06 [6.58;7.58]	7.63 [7.03;8.27]	Group	0.39	1	0.864	14.63	3	0.013
Vitamin A [µg]	RYGB	1600.12 [1264.41;2024.98]	741.59 [542.95;1012.90]	868.59 [622.50;1211.97]	731.85 [530.67;1009.30]	Meas.	42.15	3	<0.001	Meas. × Group		
	SG	1660.92 [1289.73;2138.94]	715.36 [591.24;865.54]	915.87 [721.93;1161.90]	789.60 [648.41;961.54]	Group	0.09	1	0.877	0.26	3	0.993
Vitamin E [mg]	RYGB	21.04 [17.45;25.38]	5.11 [3.88;6.72]	8.74 [5.98;12.77]	7.74 [6.06;9.89]	Meas.	224.61	3	<0.001	Meas. × Group		
	SG	21.39 [17.98;25.44]	4.94 [4.20;5.80]	7.81 [6.60;9.23]	9.48 [8.15;11.03]	Group	0.02	1	0.877	4.24	3	0.440
Vitamin B1 [mg]	RYGB	1.98 [1.71;2.29]	0.43 [0.37;0.49]	0.73 [0.59;0.91]	0.84 [0.67;1.04]	Meas.	452.26	3	<0.001	Meas. × Group		
	SG	2.54 [2.15;3.00]	0.55 [0.48;0.65]	0.72 [0.64;0.80]	0.88 [0.77;1.02]	Group	3.83	1	0.277	4.72	3	0.418
Vitamin B2 [mg]	RYGB	2.41 [2.13;2.73]	0.92 [0.81;1.05]	1.31 [1.11;1.55]	1.42 [1.19;1.70]	Meas.	218.44	3	<0.001	Meas. × Group		
	SG	2.45 [2.17;2.77]	1.02 [0.92;1.14]	1.24 [1.13;1.36]	1.28 [1.13;1.45]	Group	0.02	1	0.877	6.34	3	0.400
Niacin [mg]	RYGB	33.16 [27.94;39.36]	6.95 [5.71;8.47]	10.26 [8.28;12.71]	11.23 [8.49;14.84]	Meas.	322.81	3	<0.001	Meas. × Group		
	SG	38.90 [33.16;45.64]	11.01 [9.14;13.26]	10.98 [9.55;12.63]	12.16 [10.68;13.85]	Group	5.26	1	0.277	5.78	3	0.400
Vitamin B6 [mg]	RYGB	3.21 [2.73;3.78]	0.99 [0.87;1.13]	1.33 [1.13;1.56]	1.48 [1.22;1.79]	Meas.	278.27	3	<0.001	Meas. × Group		
	SG	4.00 [3.48;4.59]	1.18 [1.03;1.36]	1.45 [1.27;1.65]	1.54 [1.38;1.71]	Group	3.43	1	0.277	2.68	3	0.576
Folate [µg]	RYGB	318.64 [271.25;374.32]	104.80 [89.56;122.65]	140.72 [117.76;168.15]	162.35 [132.30;199.22]	Meas.	226.60	3	<0.001	Meas. × Group		
	SG	353.09 [306.04;407.38]	107.24 [93.59;122.88]	162.55 [141.82;186.31]	167.91 [148.83;189.43]	Group	0.87	1	0.864	1.91	3	0.700
Vitamin B12 [µg]	RYGB	5.42 [4.50;6.52]	2.39 [1.92;2.98]	3.22 [2.57;4.04]	3.24 [2.54;4.12]	Meas.	61.31	3	<0.001	Meas. × Group		
	SG	5.91 [5.00;6.99]	2.83 [2.30;3.48]	3.23 [2.69;3.87]	3.02 [2.54;3.59]	Group	0.28	1	0.846	3.18	3	0.532

SG, sleeve gastrectomy; RYGB, Roux-en-Y gastric bypass; Meas., measurement time; Group, surgical procedure; χ^2^, Wald chi-square statistic; *df*, degrees of freedom; *p*, *p*-value. *p*-values in Table A1 were adjusted using the Benjamini–Hochberg procedure.
